# Carcinogenic Potency of Airborne Polycyclic Aromatic Hydrocarbons in Relation to the Particle Fraction Size

**DOI:** 10.3390/ijerph15112485

**Published:** 2018-11-07

**Authors:** Gordana Pehnec, Ivana Jakovljević

**Affiliations:** Institute for Medical Research and Occupational Health, Ksaverska cesta 2, 10000 Zagreb, Croatia; gpehnec@imi.hr

**Keywords:** BaP toxic equivalency factors, particle fractions PM_10_, PM_2.5_ and PM_1_, seasonal variations, urban location, public health

## Abstract

Polycyclic aromatic hydrocarbons (PAHs) that are bound to particulate matter can have adverse effects on human health. Particle size plays an important role in assessing health risks. The aim of this study was to compare concentrations of PAHs bound to particle fractions PM_10_, PM_2.5_, and PM_1_, as well as to estimate their carcinogenic potency and relative contributions of the individual PAHs to the carcinogenic potency in relation to the size of the particle. Measurements of ten PAHs were carried out in 2014 at an urban location in the northern part of Zagreb, Croatia. 24-h samples of the PM_10_, PM_2.5_, and PM_1_ particle fraction were collected over forty days per season. Carcinogenic potency of PAHs was estimated by calculating benzo(a)pyrene equivalent concentrations while using three different toxic equivalence factor (TEF) schemes. The total carcinogenic potency (TCP) and percentage contributions differed significantly depending on the TEF scheme used. The lowest PAH mass concentrations and TCPs were in summer and the highest in winter. The contributions of individual PAHs to the sum of PAH mass concentrations remained similar in all fractions and seasons, while in fractions PM_10–2.5_ and PM_2.5–1_ they varied significantly. Road traffic represented the important source of PAHs in all fractions and throughout all seasons. Other sources (wood and biomass burning, petroleum combustion) were also present, especially during winter as a consequence of household heating. The highest contribution to the TCP came from benzo(a)pyrene, dibenzo(ah)antrachene, indeno(1,2,3,cd)pyrene, and benzo(b)fluoranthene (together between 87% and 96%) in all fractions and seasons. In all cases, BaP showed the highest contribution to the TCP regardless relatively low contributions to the mass of total PAHs and it can be considered as a good representative for assessing the carcinogenicity of the PAH mixture. When comparing the TCP of PAHs in PM_10_ and PM_2.5_ fractions, it was found that about 21–26% of carcinogenic potency of the PAH mixture belonged to the PM_2.5_ fraction. Comparison of TCP in PM_2.5_ and PM_1_ showed that about 86% of carcinogenic potency belonged to the PM_1_ fraction, regardless of the TEF scheme used.

## 1. Introduction

Airborne particulate matter represents an important public health issue. The range of harmful health effects of particulate matter is wide, and although the effects are predominantly to the respiratory and cardiovascular systems, they are not limited only to them. At present, most routine air quality measurements are focused on particle fractions PM_10_ (particles with an aerodynamic diameter smaller than 10 μm) and PM_2.5_ (particles with an aerodynamic diameter smaller than 2.5 μm), while smaller particles are still less investigated. Consequently, the majority of epidemiological studies use PM_10_ or PM_2.5_ as the exposure indicator. PM_10_ represents the particle mass that enters the respiratory tract and it includes both coarse (particle size between 2.5 and 10 μm) and fine particles (particles smaller than 2.5 μm, PM_2.5_). Coarse particles can enter the trachea (upper throat) or the bronchi. Fine particles can reach all the way down to the alveoli in the lungs [1,2,3,4,5,6] and can also penetrate from the lung alveoli into the blood circulation [7]. Smaller particles, such as PM_1_ (particles with an aerodynamic diameter smaller than 1 μm) and especially ultrafine particles (particles smaller than 0.1 μm in diameter), have recently attracted significant scientific attention and are also considered to be of importance in the context of adverse health effects induced by particulate pollution [8,9,10,11]. However, the air quality standards for particulate matter in most countries are still limited to PM_10_ and PM_2.5_ particle fraction and often exceed the WHO guideline limits [12]. 

Although particle size plays an important role in assessing health risks, it is not the only relevant factor. Many compounds that are bound to particulate matter are suspected to be genotoxic, mutagenic, and carcinogenic. They can have adverse effects to human health and can cause acute diseases [13,14,15]. Especially fine and ultrafine fractions of particulate matter may bound relatively greater amounts of toxic organic compounds when comparing to larger particles [16]. However, the toxicities of different types of particulates, individually and in mixtures, and especially the way that different components contribute to the overall toxicity of particulate matter are still poorly known [12].

Polycyclic aromatic hydrocarbons (PAHs) are organic compounds mainly produced by the incomplete combustion and pyrolysis of organic material. In urban areas, their occurrence is mostly the result of anthropogenic activities, such as traffic (vehicular, shipping, and flying), domestic heating, oil refining, waste incineration, industrial activities, agricultural activities, biomass burning, etc. [17,18,19]. Hundreds of PAHs occur in the atmosphere as complex mixtures, including PAH derivatives (such as nitro-PAHs), oxygenated products, and heterocyclic PAHs. Compounds with smaller molecular weight (2–3 aromatic rings) are present almost exclusively in the vapour-phase, whereas PAHs with higher molecular weights (more than four rings) are mostly bounded to particles [20]. PAHs are one of the first atmospheric pollutants that have been identified as suspected carcinogens. As the molecular weight of a specific PAH increases, the carcinogenicity of PAHs also increases [18,21], so the recognized carcinogenic PAHs are mostly associated with particulate matter. The US Environmental Protection Agency (EPA) classified 16 priority PAHs into two subclasses: carcinogenic and non-carcinogenic compounds. Benzo(a)pyrene (BaP) was used as a reference. These 16 priority PAHs were selected because the majority of data that related to adverse health effects referred to them [18,22]. 

Routine measurements as well as air quality standards are focused almost exclusively on PAHs (or benzo(a)pyrene, BaP, only) bound to the PM_10_ fraction. For example, Directive 2004/107/EC of the European Union set the target value of 1 ng/m^3^ for annual mean only for benzo(a)pyrene as a representative PAH (although measurements of certain other PAHs are also required). However, human exposure to PAHs is always to different types of PAHs mixtures that can have a profound effect on potency due to differences in bioavailability, carcinogenic action, or metabolism. The difficulties in dealing with guidelines for PAH mixtures using a single carcinogen indicator to represent the carcinogenic potency of a fraction of PAHs in air have been discussed previously by other authors [23]. An evaluation of BaP alone, for example, will possibly underestimate the carcinogenic potential of airborne PAH mixtures, since there are additional components contributing to carcinogenicity. A complicating factor is also that PAHs in air are bounded to particles; as particles themselves may cause adverse health effects, combined with PAHs may even enhance the carcinogenic potential of PAHs. WHO [24] does not set guideline values for genotoxic carcinogens, such as PAHs, because no safe level can be recommended, but it specifies a risk estimate for BaP in a PAH mixture as a basis for policy makers.

Several authors have used data from various cancer tests to rank the compounds according to cancer potency relative to BaP. Toxic equivalency factors (TEFs) can be used as a practical tool for large groups of compounds with a common mechanism of action, using one well known compound as a reference. For assessing the risk of PAHs in ambient air, the carcinogenic potencies of the individual PAHs are expressed relative to the potency of BaP. Carcinogenic potency of PAHs was calculated on the basis of its BaP equivalent (BaP_eq_) assessed by multiplying the concentration of an individual PAH in the air with its TEF. Different toxic equivalent factor schemes are developed by different authors, based on experiments in animals [23,25,26,27,28,29].

In the assessment of the carcinogenic potency of airborne PAHs, most studies focused on only one particle fraction. Investigations were mostly carried out on the PM_10_ or PM_2.5_ particle fractions only [4,30,31,32] and seldom on PM_1_ or smaller fractions [2,9,10]. However, previous studies comparing the PAH content of PM_10_, PM_2.5_, and PM_1_ (or smaller) fractions showed that the contribution of individual PAHs can vary significantly in different particle fractions [2,20,33,34,35,36,37,38]. Accordingly, it can be expected that carcinogenic potency will also differ significantly for different particle fractions. However, there is limited number of studies determining simultaneously the carcinogenic activity of PAHs in different particle fractions [9,20,36,39].

In this study, ten PAHs bound to fractions PM_10_, PM_2.5_, and PM_1_ were measured simultaneously at an urban location. The aim of the study was to compare the PAH carcinogenic potency in different particle fractions and estimate the contribution of individual PAHs to the total carcinogenic activity of the PAH mixture. The carcinogenic potency of PAHs in this study was calculated using different TEF factors from literature [27,28,29] in order to see whether the use of different factors would cause statistically significant differences in the estimation of PAH carcinogenic activity among different particle fractions. All analyses were carried out for the overall period and for each season separately, as well.

## 2. Materials and Methods

**Figure 1:**
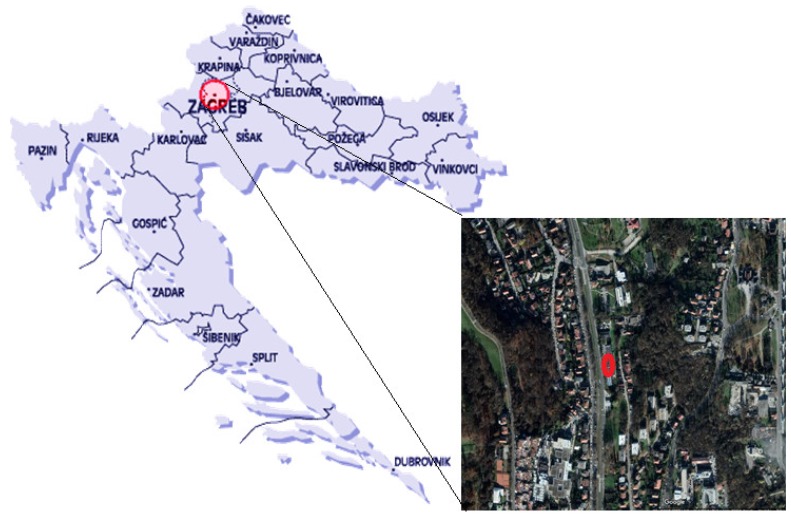
Position of the measuring site in Zagreb.

The measuring site was located in the northern, residential part of Zagreb, the Croatian capital (45°50′6.83″ N, 15°58′42.12″ E, 168 m a.s.l.) at the approximate distance of 50 m from the nearby road with modest traffic density (Figure 1). The site is surrounded mostly with family houses which use both natural gas and wood furnaces for domestic heating purposes. The climate is continental, and the heating season usually lasts from October to April. Three fractions of particles, PM_10_, PM_2.5_ and PM_1_ were collected simultaneously on quartz filters with a low volume Sven Leckel sampler from about 55 m^3^ of air. The samplers were set at about 1.5 m above the ground. 24-h samples of particulate matter were collected over forty days per each season during the year 2014. Sampling periods were: 1 January–22 February (winter), 22 March–11 May (spring), 24 June–7 August (summer), and 27 September–9 November (autumn). A total amount of 160 samples was collected for each particle fraction. 

After collection, samples were wrapped in aluminium foil and kept in a deep freezer at −18 °C until analysis [40]. Filters were extracted with a solvent mixture of toluene and cyclohexane (7:3) in an ultrasonic bath for one hour, separated from undissolved parts by centrifugation (10 min, 3000 rpm), and evaporated to dryness in a mild stream of nitrogen at 30 °C. They were then re-dissolved in acetonitrile. The PAH analysis was performed using a Varian Pro Star high performance liquid chromatography (HPLC) with a fluorescence detector (FLD) and programmed changes in excitation and emission wavelength. Samples were analysed for the following ten PAHs: fluoranthene (Flu), pyrene (Pyr), benzo(a)antrachene (BaA), chrysene (Chry), benzo(b)fluoranthene (BbF), benzo(k)fluoranthene (BkF), benzo(a)pyrene (BaP), dibenzo(ah)antrachene (DahA), benzo(ghi)perilene (BghiP), and indeno(1,2,3,cd)pyrene (IP). PAHs were separated on a Varian stainless steel Pursuit 3 PAH column (100 × 4.6 mm). The mobile phase was a mixture of acetonitrile and water (60:40), and the flow rate was 0.55 mL min^−1^. For every group of filters, laboratory and field filter blank were also prepared. Laboratory and field blanks were processed the same way as the collected samples. The HPLC/FLD system was calibrated using the Supelco EPA 610 PAHs Mix. Standard working calibration solutions were obtained by diluting certified solutions with Merck HPLC-grade acetonitrile. The chromatogram of blanks showed no peak at retention time specific for the analyses PAHs. For that reason, the method detection (DL) and quantification limits (QL) for each PAH were calculated as concentration equivalents to three (DL) and ten times (QL) the signal-to-noise ratio. The detection of PAHs was based on the measurements of emitted fluorescence after the excitation with specific wavelength. As the excitation and emission wavelengths differed for different PAHs, the signal to noise ratio was also different at the retention times specific for each PAH. Signal to noise ratios were then recalculated to mass concentrations by using corresponding calibration curves. QL ranged from 0.007 ng m^−3^ for BaA to 0.18 ng m^−3^ for Flu.

The accuracy of the method was determined by analysing the analytical standard (Supelco EPA 610 PAH mix) and the certificate reference material (CRM NIST 1649b, Urban dust). Recovery of PAHs from the certificate reference material ranged from 72.5% for Flu to 110.2% for BghiP. The detailed analytical procedure is described in Jakovljević et al. [41].

The carcinogenic potency of PAHs was estimated on the bases of BaP equivalents. BaP_eq_ were calculated by multiplying mass concentration of individual PAH with its respective toxic equivalency factors. In this study, three different TEF schemes were used, as proposed by Nisbet and LaGoy [27], Muller [28], and Larsen and Larsen [29]. Those TEFs are presented in Table S1 of the Supplementary Materials. To express the carcinogenicity of the mixture, total carcinogenic potency (TCP) was calculated by summing up the BaP_eq_ of each measured PAH. The calculation was carried out according to the Equation (1):

TCP = ƩBaP_eq_(PAH) = ƩTEF(PAH) × γ(PAH) (1)

TCP—total carcinogenic potency

TEF—toxic equivalency factors of particular PAH

γ—mass concentrations of particular PAH

Relative potency factor (RPF) was determined as the ratio between the TCP (sum of all BaP_eq_) to the concentration of BaP, according to the Equation (2):

RPF = TCP/γ (BaP)(2)

RPF—relative potency factor

TCP—total carcinogenic potency

γ (BaP)—measured mass concentration of BaP

The percentage contribution of the carcinogenic potency of individual PAHs to the total carcinogenic potency was calculated as well. Calculations were carried out for all three particle fractions, for the overall period, and for each season separately. 

The results were statistically treated by Statistica software, version 13.2 (Dell Inc., Round Rock, TX, USA). 

## 3. Results 

### 3.1. Mass Concentrations of PAHs in PM_10_, PM_2.5_ and PM_1_ Particle Fractions

Mass concentrations of ten PAHs bound to PM_10_, PM_2.5_, and PM_1_ particle fraction were measured over forty days per season at the urban location in Zagreb throughout 2014. The sum of PAHs (ΣPAH) was calculated for each day. The range, mean, standard deviation, and median value of 24-h PAH mass concentrations are presented on a seasonal basis in Table 1, Table 2 and Table 3 for PM_10_, PM_2.5_, and PM_1_, respectively. From Table 1, Table 2 and Table 3 it is obvious that the highest mass concentrations were measured in winter and the lowest in summer. In all three fractions and through all seasons BhgiP presented the highest concentrations and DahA the lowest.

**Figure 2:**
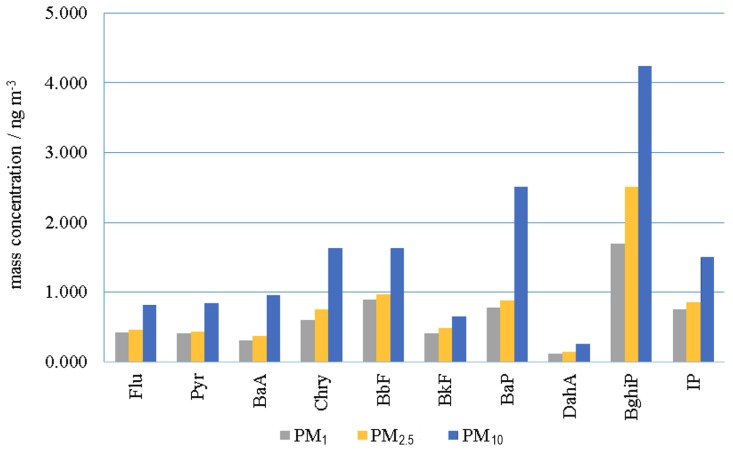
Average polycyclic aromatic hydrocarbons (PAH) mass concentrations in PM_10_, PM_2.5_, and PM_1_ particle fraction during 2014 at an urban location in Zagreb.

Average PAH concentrations (arithmetic mean) for the overall period for all three fractions are shown in Figure 2. The average ΣPAH (and corresponding standard deviations) for the whole measuring period were 15.009 ± 22.061, 7.824 ± 9.798, and 6.364 ± 8.055 ng m^−3^ for the PM_10_, PM_2.5_, and PM_1_ fraction, respectively. The sum of PAHs in 24-h samples was in range 0.055–124.386, 0.165–44.342, and 0–34.709 ng m^−3^ for the PM_10_, PM_2.5_, and PM_1_ fraction, respectively. In PM_2.5_ and PM_1_ particle fractions average PAH concentrations followed very similar order: BghiP, BbF, BaP and IP showed the highest concentrations and Pyr, BaA, and DahA the lowest. In PM_10_ fraction, the order was slightly different: BghiP, BaP, and Chry showed the highest concentrations and Flu, BkF, and DahA the lowest.

**Figure 3:**
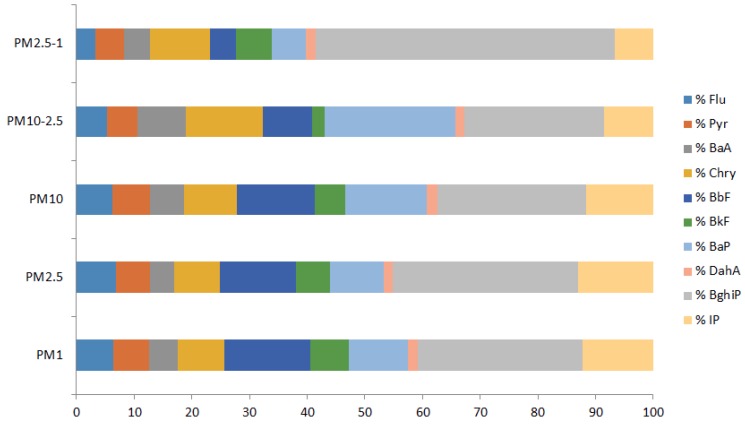
The percentage contribution of individual PAHs to the sum of measured PAHs (the overall measuring period).

The average percentage contribution of the particular PAH to the sum of total PAHs is shown on Figure 3. The same is presented for each season separately in Supplementary Materials, Figure S1. In the PM_10_ particle fraction, BghiP showed the highest percentage contributions of PAHs, followed by BaP and BbF. DahA showed the lowest percentage contribution. The average BaP contribution in the PM_10_ fraction (and corresponding standard deviation) was 14 ± 4% for the overall period (Figure 3). The average BaP contribution in PM_10_ was the lowest in autumn (11 ± 2%) and the highest (18 ± 3%) in winter (Figure S1). In the collected 24-h samples, the contribution of BaP varied between 3.4% to 31.4%, depending on meteorological conditions and dominant sources during a particular day. In both the PM_2.5_ and PM_1_ fractions, the highest contribution originated from BghiP, followed by BbF and IP. The average percentage contributions of BaP (and their standard deviations) for the overall period were similar in those two fractions: 9 ± 4% and 10 ± 4% in PM_2.5_ and PM_1_, respectively (Figure 3). The average contribution of BaP to the sum of PAH in the PM_2.5_ fraction was between 5 ± 3% and 12 ± 2%, depending on the season, while in the PM_1_ fraction it was between 9 ± 2% and 13 ± 3% (Figure S1). However, in the daily samples, BaP contribution varied between 0–18% and 0–23% for the PM_2.5_ and PM_1_ particle fraction, respectively. The contributions of PAHs in subtracted fractions PM_10–2.5_ and PM_2.5–1_ differed significantly between seasons (PM_2.5–1_ fraction was calculated by subtracting PM_1_ from PM_2.5_, and PM_10–2.5_ by subtracting PM_2.5_ from PM_10_). The average percentage contribution of BghiP in the coarse fraction, PM_10–2.5_, varied from 6% in summer to 26% in winter, while average BaP contribution ranged from 13% in autumn to 26% in summer and winter. In summer and autumn, the highest contribution in that fraction showed BbF, while in other seasons it was much lower (about 6%). Regarding the fraction PM_2.5–1_, the differences were even more pronounced for BghiP: its average contribution varied from 67% in winter to about 20% in spring and autumn. Average BaP contribution ranged from 1% (summer) to 12% (autumn). In summer, the highest contribution showed IP (42%), while in all other seasons its contribution was between 4 and 13%. The average contributions of BbF in spring and autumn were much higher than in winter and summer.

Relationships between individual PAHs in different particle fractions were presented by the linear regression Equation (3):

(PAH)_PMy_ = *a* × (PAH)_PMx_ + *b* (3)

where *a* is the slope of the regression line, *b* the intercept of the regression line, (PAH) is the mass concentration of individual PAHs in the PM_x_ and PM_y_ particle fractions. Such linear regression analysis was carried out in order to determine the quantity of PAH in the smaller (PM_y_) fraction as compared to the larger (PM_x_) fraction (the slope of the regression line represents the quantity of PAH in PM_y_ fraction compared to PM_x_). The procedure is described in more detail in Jakovljević et al. [42]. Due to the fact that PM_1_ is contained in PM_2.5_, and PM_2.5_ in PM_10_ particle fraction, variables (PAH)_PMx_ and (PAH)_PMy_ are dependent and statistically significant correlation is expected. However, to obtain more information about the relationship between fractions, the same analysis was also carried out between PM_1_ and PM_2.5–1_ and PM_2.5_ and PM_10–2.5_ particle fractions (PM_2.5–1_ fraction was calculated by subtracting PM_1_ from PM_2.5_, and PM_10–2.5_ by subtracting PM_2.5_ from PM_10_). The slopes and the intercepts of linear regression lines together with the corresponding correlation coefficients (*r*) for the whole measuring period are presented in Table 4. The correlations between the sum of PAH mass concentrations in different particle fractions are presented graphically in Figure S2 of the Supplementary Materials.

The correlations obtained between PAHs in PM_1_ and PM_2.5_ particle fractions were much stronger than between PM_10_ and PM_2.5_, with corresponding correlation coefficients between 0.672–0.968 and 0.492–0.715 (*p* < 0.05), respectively (Table 4). The slopes of the regression line for the overall period indicated that only between 18% (BaA) and 40% (BkF) of the PAHs measured in PM_10_ were present in the PM_2.5_ fraction, and about 29% of total PAHs in PM_2.5_ were present in the PM_10_ fraction (*r* = 0.660) (Figure S2). When comparing the ratio of PAH mass concentrations in particle fractions PM_2.5_ and PM_1_, it is evident that between 68% and 85% of PAHs measured in PM_2.5_ were present in the PM_1_ fraction (except for BghiP and DahA, which were present 55 and 59%, respectively). Comparison of ΣPAH in PM_2.5_ and PM_1_ showed that about 79% of total PAHs in PM_2.5_ were present in the PM_1_ fraction (*r* = 0.968). Obtained ratios showed that, for the overall period, most of the PAHs were present in larger particles. 

The correlations obtained between PAHs in PM_1_ and PM_2.5–1_, and PM_2.5_ and PM_10–2.5_ particle fraction were much lower than those between PM_1_ and PM_2.5_ or PM_2.5_ and PM_10_ (which was expected due to the fact that PM_1_ is contained in PM_2.5_, and PM_2.5_ in PM_10_), although all significant (except for BkF between the PM_2.5_ and PM_10–2.5_ fractions). The correlation coefficients between PAHs in PM_1_ and PM_2.5–1_ were in the ranges of 0.43–0.68 and the highest for BghiP, Pyr, and BaA. Comparing PAHs in the PM_10–2.5_ and PM_2.5_ particle fraction it was found that correlation coefficients ranged between 0.13 and 0.65. The highest correlation coefficients were found for BghiP, BaP and IP. High correlation coefficients of these regression lines may indicate the common sources, however the slopes do not indicate contributions because the aforementioned fractions are mutually independent. 

### 3.2. Carcinogenic Potency of PAHs Bound to Different Particle Fractions

BaP equivalents were calculated for each PAH using TEFs that were published previously by Nisbet and LaGoy [27], Muller [28], and Larsen and Larsen [29]. Total carcinogenic potency and relative potency factor were calculated per day, as well as the percentage contribution of BaP equivalents of individual PAH in the TCP. Mean TCP and RPF values and their standard deviations per each season and for the whole period are presented in Table 5 and Figures S3–S4 of the Supplementary Materials. The highest TCPs and RPFs were obtained when the TEF of Nisbet and LaGoy [27] were used, and the lowest when the TEFs of Muller [28] were applied. In all cases, TCPs followed the order winter > autumn > spring > summer. RPFs in PM_10_ were the highest in autumn and similar in spring and summer. RPFs in PM_2.5_ were the highest in spring and similar in autumn and winter, while RPFs in PM_1_ were the highest in spring, and similar in all other seasons.

Student *t*-test of depended samples was used to see whether the differences between results were statistically significant when different TEF schemes were applied. Although the TCPs and RPFs obtained with the TEFs of Muller [28] and Larsen and Larsen [29] seem similar (Table 5), for all seasons and all fractions it was found that TCP values differed significantly between groups with different TEF schemes (except in summer in PM_2.5_ fraction). The percentage contributions of PAHs in TCP also differed significantly between groups with different TEF schemes (except for IP in all four seasons, all fractions, and DahA in summer in PM_2.5_ and PM_1_ fractions). RPF values also differed significantly between groups with different TEF schemes, except in summer in the PM_2.5_ fraction. However, high linear correlation coefficients were found between TCP and RPF values (Table S2 of the Supplementary Materials). Correlation coefficients were between 0.9965 and 0.9999 for TCP and between 0.8542 and 0.9732 for RPF. 

The percentage contributions of a particular PAH to the total TCP for each particle fraction, season, and TEF scheme are presented in Tables S3–S5 of Supplementary Materials. Regardless of the TEF scheme used, the highest contribution to the total carcinogenic potency was from BaP, through all seasons and for the overall period and for all fractions. The only exception was for the PM_1_ fraction during spring, when the percentage contribution of DahA was slightly higher when the TEFs of Nisbet and LaGoy [27] were applied. This comes as no surprise, because due to the high TEF for DahA (5.0) the small changes in DahA/BaP mass concentration ratio may cause significant changes in DahA contribution to the TCP. When TEFs of Nisbet and LaGoy [27] were used, the BaP carcinogenic contribution ranged from 40% (spring) to 58% (summer) for PM_1_ particle fraction, from 42% (spring) to 48% (summer) for PM_2.5_, and from 48% (autumn) to 62% (winter) for PM_10_ particle fraction. Estimation of BaP contribution to TCP with TEFs from Muller [28] gives much higher values: from 62% (spring) to 71% (winter) for PM_1_ particle fraction, from 55% (summer) to 69% (autumn and winter) for PM_2.5_, and from 68% (autumn) to 82% (winter) for PM_10_ particle fraction. Similar values were obtained when the TEFs of Larsen and Larsen [29] were used: from 56% (spring) to 66% (winter) for PM_1_ particle fraction, from 49% (summer) to 64% (winter) for PM_2.5_ and from 65% (autumn) to 78% (winter) for PM_10_ particle fraction. In all cases (Tables S3–S5), the compounds with the highest contribution to TCP were BaP, DahA, IP and BbF. Regardless of the TEF scheme, the total contribution of those four compounds together to the TCP was between 87% and 96% in all fractions and through all seasons, regardless of the selected TEF scheme. The highest percentages were obtained with TEFs of Muller [28]. All other PAHs individually contributed less than 5% in all fractions and seasons. The differences in contribution of each individual PAH to the TCP were smaller between fractions than between seasons. 

**Figure 4:**
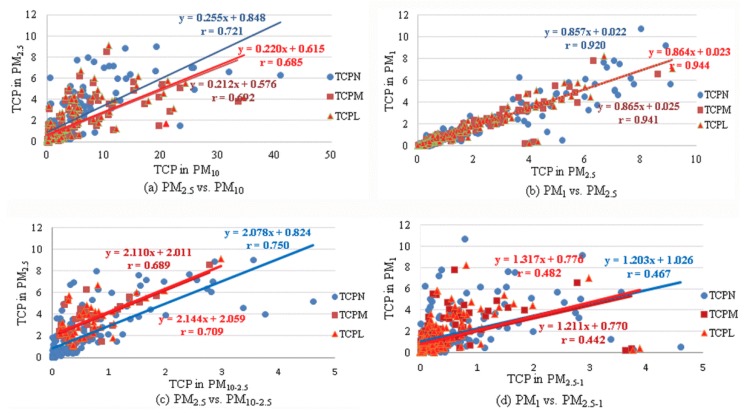
Correlation between total carcinogenic potencies, TCP (ng m^−3^) in particle fractions: (**a**) PM_2.5_ and PM_10_; (**b**) PM_1_ and PM_2.5_; (**c**) PM_2.5_ and PM_10–2.5_; (**d**) PM_1_ and PM_2.5–1_, for the overall measuring period; TCP_N_—total carcinogenic potency calculated using toxic equivalency factors of Nisbet and LaGoy [27]; TCP_M_—total carcinogenic potency calculated using toxic equivalency factors of Muller [28]; TCP_L_—total carcinogenic potency calculated using toxic equivalency factors of Larsen and Larsen [29].

The correlation between total carcinogenic potencies that were determined for different particle fractions was analysed as well. The same methodology that was used for the determination of the correlation between individual PAHs in different particle fractions was applied. The parameters *a* and *b* of linear regression equation (TCP)_PMy_ = *a* × (TCP)_PMx_ + *b* and corresponding correlation coefficients were determined between the TCP values in different fractions (Figure 4). 

The slopes of the linear regression lines from Figure 4a,b indicate how much of the carcinogenic potency of smaller fraction is present in the larger fraction. It was found that about 21–26% of carcinogenic potency of the PAH mixture in PM_10_ is present in the PM_2.5_ fraction (Figure 4a). The highest slope was obtained when the TEFs of Nisbet and LaGoy [27] were used, while for the TEFs of Muller [28] and Larsen and Larsen [29] they were almost identical. Comparison of TCP between PM_2.5_ and PM_1_ (Figure 4b) showed that about 86% of the carcinogenic potency of PM_2.5_ is present in the PM_1_ fraction, and the same result was obtained, regardless of the TEF scheme used. The correlation coefficients of all linear regression lines were significant and ranged between 0.685 and 0.944, which proves that the linear regression model is appropriate for the comparison of TCP in different fraction. Correlations presented in Figure 4c (between TCP in PM_2.5_ and subtracted value PM_10–2.5_) and Figure 4d (between TCP in PM_1_ and subtracted value PM_2.5–1_) indicate the relationship between TCPs in independent fractions. The correlation coefficients were smaller than those presented on Figure 4a,b, although all significant. Again, better correlation was found between smaller fractions—PM_1_ and PM_2.5−1_ (r between 0.689 and 0.750, Figure 4d) than between PM_2.5_ and PM_10−2.5_ (*r* between 0.442 and 0.482, Figure 4c).

**Figure 5:**
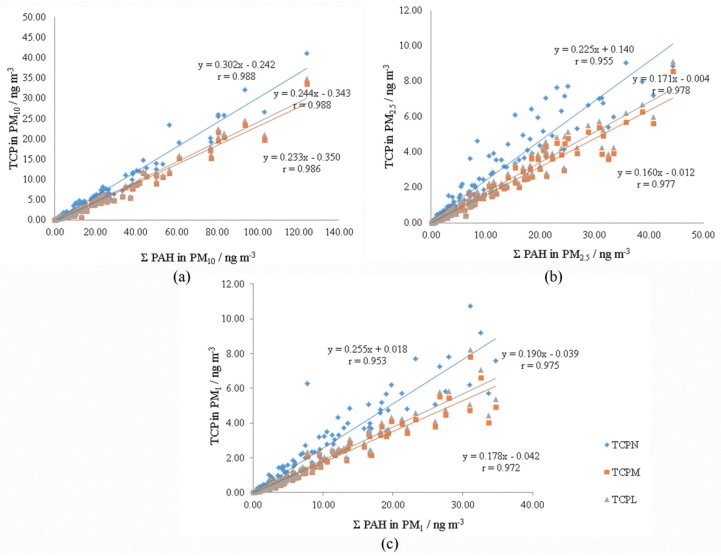
Correlation between total carcinogenic potencies (TCP) and the sum of PAH mass concentrations (ΣPAH) in particle fractions: (**a**) PM_10_; (**b**) PM_2.5_; and (**c**) PM_1_ (the overall measuring period).

In further analysis the correlation between TCP and the sum of PAH mass concentrations, ΣPAH, was analyzed for each particle fraction separately. Scatter plots with linear regression lines between TCP and ΣPAH and corresponding correlation coefficients are presented in Figure 5. For all fractions the slopes of regression lines were similar when TCPs were calculated while using TEFs of Muller [28] and Larsen and Larsen [29], while slopes obtained with TEFs of Nisbet and LaGoy [27] were 24 to 42% higher. Regardless of the TEF scheme used, the slopes were the highest for PM_10_ and the lowest for PM_2.5_ particle fraction. From the regression lines it can be estimated that an increase of ΣPAH of 10 ng m^−3^ led to the increase in TCP between 2.3 and 3.0 ng m^−3^, while the same increase of ΣPAH in PM_2.5_ fraction increased TCP between 1.6 and 2.2 ng m^−3^.

## 4. Discussion

### 4.1. Mass Concentrations of PAHs in the PM_10_, PM_2.5_, and PM_1_ Particle Fractions

Mass concentrations of ten PAHs bound to the PM_10_, PM_2.5_, and PM_1_ particle fraction measured in this study, as well as ΣPAH showed characteristic variations with the highest values during winter (heating season). Šišović et al. [40], Godec et al. [43], and Jakovljević et al. [41] were determined the similar seasonal differences at the same location as in this study, but their studies were carried out in 2008, 2010, and 2010/2011, respectively. However, all of these studies were limited only to the PM_10_ particle fraction. Average mass concentrations of PAHs in this study as well as in the aforementioned studies followed the similar order in all seasons, with the highest average concentration for BghiP and the lowest for DahA. 

A comprehensive study carried out in 2013 at the same location in Zagreb showed that there were differences in PAH concentrations bound to the PM_10_, PM_2.5_, and PM_1_ particle fractions between seasons, which was found to be related with the differences in contribution of individual pollution sources. The main PAH sources were household heating, traffic (diesel and gasoline burning), and wood burning. The selected location was found to be a good representative for the wider urban area [42]. The study by Jakovljević et al. [42] focused on PAH mass concentrations, the relationship between PAHs in different fractions, relationship with meteorological conditions, and the determination of potential pollution sources. However, in that study, certain other important issues were not addressed, especially those related to the estimation of possible harmful health effects. The aim of this study was to focus primarily on the estimation of carcinogenic activity of PAHs in different fractions. Rather than to re-analyze already published data from 2013 [42], we have included new unpublished measuring data from 2014, hoping that they will also give us insight into the variations of PAH between years. The PAH concentrations that were measured in this study in the PM_10_ fraction are slightly higher than those obtained in the studies of Jakovljević et al. [42] and Šišović et al. [44] at the same location. On the other hand, concentrations of PAHs in PM_2.5_ and PM_1_ in this study were lower as compared to those carried out in the previous year [42]. One of the possible reasons could be meteorological conditions, especially during heating season: temperature and relative humidity in this study were higher than in 2013 (for example, the average temperature during winter in this study was 5 °C, as compared to 2.5 °C in the study of Jakovljević et al. [42]). Meteorological conditions during the four measuring periods of this study are presented in Table S6 of the Supplementary Materials. The other possible reason is the fuel used in the households for heating purposes. Although most houses in the area introduced natural gas about twenty years ago, due to economic reasons in colder years many of them switched to wood furnaces. The results of this study show that differences in PAH levels between years exist in all three particle sizes. However, for a better understanding of PAH trends and intra-annual variabilities, the measurements should continue in the future.

Regardless of the obtained differences in PAH concentrations between years, all of the measured values were still within the range measured in other urban regions in Europe [4,21,33,35,45]. At some locations, slightly lower concentrations of PAHs in PM_10_ and PM_2.5_ fractions were observed [4,35,45]. As for the PM_1_ particle fraction, Rogula-Kozlowska et al. [33] and Kozielska et al. [10] found much higher concentrations of PAHs in Poland. In the study of Kozielska et al. [10] during heating season, the highest concentrations at the urban background site were noted for BaA, Flu, Chry, BaP and Pyr (ΣPAH 23.1 ng m^−3^), while at the urban traffic location the highest concentrations showed Flu, DahA, Chry, Pyr, BaA, BkF, BbF, and BaP (ΣPAH 186.1 ng m^−3^). In our study, the concentrations of Flu, BaA, and DahA were much lower as compared to other PAHs (e.g., BghiP, BbF) and indicate different sources of pollution.

The relative contributions of the individual PAH to the sum of total PAHs in this study varied slightly, depending on the season. For the overall period BghiP, BbF, BaP, and IP together contributed 66%, 67%, and 66% for the PM_10_, PM_2.5_, and PM_1_ fraction, respectively. The contribution of these four PAHs remained similar through all seasons, varying between 62% and 72%. In summer, in the PM_2.5_ fraction, the percentage contribution of BghiP was the highest (38%) and the contribution of BaP was the lowest when comparing to other seasons. High contributions of PAHs with higher molecular weights indicated traffic as a possible pollution source of PAHs, as those PAH compounds are often present in exhaust gases [38]. The average BaP contribution in PM_10_ ranged between 11% (autumn) and 18% (winter), while in PM_2.5_ it was between 5% (summer) and 12% (autumn). The contribution of BaP to the sum of PAH in the PM_1_ fraction was between 8% (spring) and 12% (winter), depending on the season, which is similar to the results obtained by Kozielska et al. [10] in Poland, where BaP contributed 9–13%. However, percentage contributions of Flu and Pyr were higher than those that were measured in this study in Zagreb, probably as a result of the more widespread use of wood and coal during heating season in Poland. More information regarding pollution sources can be obtained by analyzing the contributions of individual PAHs in PM_10–2.5_ and PM_2.5–1_ fractions. Contrary to what has been found for PM_10_, PM_2.5_, and PM_1_ fractions in this study, where the contributions of individual PAHs to the sum of PAH mass concentrations remained similar in all fractions and seasons, the contributions of PAHs in subtracted fractions varied a lot. For the overall measuring period in the PM_10–2.5_ fraction the highest average percentage contributions showed BghiP and BaP. However, the average percentage contribution of BghiP varied from 6% in summer to 26% in winter, while average BaP contribution ranged from 13% in autumn to 26% in summer and winter. In summer and autumn, the highest contribution in that fraction showed BbF, while in other seasons it was much lower. In summer, high BbF contribution combined with relatively low contribution of BaP indicate traffic as a possible source of PAHs in coarse fraction. Regarding of the fraction PM_2.5–1_, the differences were even more pronounced for BghiP (from 20% to 67%). BaP contribution (1–12%) was lower as compared to the PM_10–2.5_ fraction. In summer, the highest contribution showed IP, while in all other seasons its contribution was low. The average contribution of BbF in spring and autumn was much higher than in winter and summer. High contribution of BghiP in winter followed by low contribution of IP indicate petroleum combustion, while low BaP /BghiP contribution ratio indicate traffic as a PAH source in PM_2.5–1_ fraction [41]. In a study by Jakovljević et al. [42], the PAH contributions to the overall mass of particles (percentage mass ratio PAH/PM) were calculated for all fraction. These contributions did not change significantly throughout the year, and although they are not directly comparable with the contributions calculated in this study, they lead to the same general conclusion: in both studies, traffic was indicated as a possible sources of PAHs in PM_1_ during summer, while during autumn and winter the dominant source of PAHs in PM_10_ and PM_2.5_ was house heating. 

Correlations between individual PAHs in different particle fractions were investigated using linear regression analysis. All of the obtained correlation coefficients were statistically significant (*p* < 0.05), which, together with relatively small intercepts, shows that the linear regression model is an appropriate tool for the determination of PAH concentration ratios between the different particle fractions. Comparison of PAHs in the fractions PM_10_, PM_2.5_ and PM_1_ for the overall period (Table 4) showed that between 18% and 40% of PAHs measured in PM_10_ were present in the PM_2.5_ fraction, while between 67% and 85% of PAHs measured in PM_2.5_ were present in the PM_1_ fraction (ratios were lower for BghiP and DahA; PAHs that probably originate from car exhaust [21]). From the obtained percentage ratios, it is evident that for the overall period most of the PAHs were bonded to larger particles. In urban areas, PM_10_ are mostly generated from different incomplete combustion processes and diesel vehicles represent the important source of high-weight PAHs, such as BghiP and DahA in coarse particles [21]. The correlation coefficients between PAHs in PM_2.5_ and PM_10–2.5_ for the overall period (Table 4) also indicate that traffic-related PAHs were mostly bonded to larger fractions of particles: the high-weight PAHs with five and six aromatic rings (BghiP, IP, and BaP, specific for car exhausts [21]) showed the stronger correlation as compared to the low-weight PAHs. Comparing the smaller fractions (PM_1_ and PM_2.5–1_), the strongest correlation showed Pyr, BaA and BghiP, PAHs characteristic for processes such as wood burning and petroleum burning [20,21,38]. The results indicate that the common source of PAHs in both smaller fractions (PM_2.5_ and PM_1_) of particulate matter is household heating. In other studies in Europe, higher contributions of PAHs in PM_2.5_ related to the PM_10_ fraction were found. For example, Andreou et al. [35] found that more than 98% of the identified PAH compounds were bound to PM_2.5_ rather than PM_10_ particles. The investigation of PAHs in traffic tunnels found that 95% of total PAHs were associated with a fraction smaller than 1 µm [46]. However, these locations were exposed to much higher traffic when compared to this study. The low ratios of PAHs in PM_2.5_ compared to the PM_10_ in this study show a high presence of PAHs in larger particles and may be connected with incomplete combustion in car exhausts due to the age of vehicle or may indicate PAH sources other than traffic (diesel, biomass burning).

In general, the descriptive statistics, linear regression analysis and percentage contribution analysis of PAH mass concentrations carried out in this study showed that PAH levels and annual variations were similar to the results that were obtained in some other European cities [4,21,33,35,45]. Traffic represented the most important source of PAHs in all fractions and throughout all seasons. Other sources were also present (wood and biomass burning) especially during winter. The contribution of BhgiP, IP (6-ring PAHs), BaP and BbF (5-ring PAHs) to the total PAHs in PM_10_, PM_2.5_, and PM_1_ remained similar throughout all seasons and fractions. The contribution of BaP that was obtained in this study was similar to the contribution published previously [10].

### 4.2. Carcinogenic Potency of PAHs Bound to Different Particle Fractions

The total carcinogenic potency of PAHs bounded to particle fractions PM_10_, PM_2.5_, and PM_1_ in this study was estimated using the toxic equivalency factors (TEFs) of Nisbet and LaGoy [27], Muller [28] and Larsen and Larsen [29]. Nisbet and LaGoy [27] reviewed earlier relative potency estimates and provided revised ones. Their estimation of TEFs was based on studies that included carcinoma appearances in lungs of rats exposed via intrapulmonary administration, complete carcinogenesis in mouse skin, papillomas, and/or carcinomas on mouse skin in initiation-promotion studies, sarcomas at the site of injection following subcutaneous administration to mice, and PAH–DNA adducts in in vitro studies [23,27]. TEF values by Muller [28] were based primarily on tumour initiation in mouse skin. If such data were lacking, data from assays on rat lungs or complete carcinogenicity data from mouse skin were used. The data were compared at a standardized time of observation. The TEFs of Muller [28] are mostly in good agreement with those by Nisbet and LaGoy [27], except for DahA (the value by Nisbet and LaGoy is five times higher) [23,28]. The Larsen and Larsen [29] TEF scheme is based on the database on carcinogenicity studies using various routes of administration (oral, pulmonary, and skin application) [23,29]. The TEF values are quite similar to other two aforementioned schemes; however, the TEF for Flu is 0.05 as compared with 0.001 (Table S1) [27,29], which may cause great differences, as Flu occurs at relatively high levels in ambient air at some locations [10]. In addition, BaA has lower TEF values by Larsen and Larsen [29] (0.0005) than by Nisbet and LaGoy [27] (0.01). That led to the conclusion that TEF values should be selected considering the type of location, potential pollution sources, and atmospheric conditions. It is difficult to recommend which TEF scheme should be used at which location. However, in future research authors should take into account that at locations with high levels of DahA (e.g., urban locations exposed to traffic or gasoline burning) carcinogenic potency of PAHs will probably be overestimated when the TEFs of Nisbet and LaGoy [27] are applied. For that reason, some authors use Nisbet and LaGoy’s scheme but with a TEF value of 1.0 for DahA (as suggested by some other authors [23]) instead of 5.0. On the other hand, at locations with high levels of Flu (exposed to coal and biomass burning) the use of Larsen and Larsen [29] TEF scheme will also result in the overestimation of total carcinogenic potency.

Carcinogenic potency of PAHs was determined previously in Zagreb in an older study that was carried out in summer 2010 and winter 2011 [41], and it included only the PM_10_ particle fraction. In this study, the calculated total carcinogenic potencies, relative potency factors and the percentage contributions of particular PAH in TCP differed significantly depending on the TEF scheme used, although strong correlation was found between results. The highest values were obtained when TEFs by Nisbet and LaGoy [27] were used, probably as a consequence of the high TEF value for DahA. TCPs calculated with TEFs by Muller [28] and Larsen and Larsen [29] were more similar. In a study by Delgado-Saborit et al. [47], different TEF schemes were also used (Table 6) for PAH mass concentrations determined at traffic roadside; however, the authors did not find significant differences in average TCP and percentage contributions of individual PAHs. On the other hand, Ayoko et al. [48] measured PAH concentrations at urban site around Brisbene, Australia, and concluded in their study that the TEF scheme might significantly influence the estimation of cancer risk. Total carcinogenic potencies observed by other authors at different locations worldwide are shown in Table 6. However, the comparison of those results with this study is difficult due to the following reasons: PAHs were determined in different particle fractions or in both particle and gaseous phase; the number of PAHs investigated ranged considerably (for example, it amounted to eight in a study by Jung et al. [49], and 88 in a study by Samburova et al. [50]); different TEF schemes were used; different numbers of samples were collected with different distributions over the year. The TEFs by Nisbet and LaGoy [27] seem to be used the most often, although many authors used the modified version with a TEF value of 1.0 for DahA instead of 5.0. However, some studies were more similar to this study regarding the measured particle fraction and TEF schemes applied [9,10,47]. Results from the study of Pooltawee et al. [9] and Kozielska et al. [10] were higher than the results that were obtained in this study, especially during the heating period. Carcinogenic potencies determined using a modified Nisbet and LaGoy scheme were similar or slightly lower than TCPs calculated in this study at some similar locations, for PM_10_ [30,32,39,51], PM_2.5_ [4,31,39,49], and PM_1_ [2] particle fraction. 

The percentage contributions of a particular PAH to the TCP calculated for each particle fraction and each season showed that the highest contribution to the total carcinogenic potency showed BaP, regardless of the TEF scheme used. The BaP carcinogenic contribution ranged from 40% (spring, PM_1_ particle fraction) to 62% (winter, PM_10_ particle fraction) when TEFs by Nisbet and LaGoy [27] were used. An estimation of BaP contribution to TCP with TEFs from Muller [28] gives much higher values: from 55% (summer, PM_2.5_) 82% (winter, PM_10_). Similar contributions were obtained when TEFs by Larsen and Larsen [29] were used: from 49% (summer, PM_2.5_) to 78% (winter, PM_10_). In all cases, the highest contributions to TCP were from BaP, DahA, IP, and BbF (while the most abundant compounds were BghiP, BbF, BaP, and IP, see Figure 3 and Figure S1 of Supplementary Materials). When comparing the contribution of BaP mass concentrations to the ΣPAH concentrations (between 5% and 18%) with its contribution to the TCP, it is evident that, even in cases when BaP concentrations were low, it had a strong contribution to the carcinogenic potency. The strongest contribution of BaP to the TCP was in winter in PM_10_ (regardless of the TEF scheme and the fraction) accompanied by the lowest contributions of BbF, BkF, and IP to the TCP. The ratio of mass concentrations BaP/(BbF + BkF + IP) was the highest in winter and after multiplying measured mass concentrations with the corresponding TEFs it pointed even more to the BaP_eq_ and the contribution to TCP. Relatively higher mass concentrations of BaP in winter as compared to BkF, BbF, and IP are characteristic for the heating season and the consumption of solid fuels, such as coal, wood, or biomass. Similar contributions were found in the study by Delgado-Saborit et al. [47]. The highest contribution was recorded for BaP (54 ± 17%), followed by DahA (20 ± 20%) and BbF (9 ± 6%). These results are consistent with carcinogenic profiles that were reported in the literature where BaP is the main contributor to the overall carcinogenic potency of the PAH mixture [47]. Furthermore, the percentage contribution of BaP in TCP in winter at urban locations in Thessaloniki, Greece [39] was 57% and 48% for the urban-traffic and the urban background site, respectively. In the warm period, the contribution of BaP decreased slightly at the traffic site (44%) but drastically at the urban background site (1%). Jang et al. [49] determined the TCP in New York City by measurements of eight PAHs in PM_2.5_ and gaseous phase and also found that BaP, DahA, BbF, and IP showed the highest percentage contribution to TCP. Overall, the analysis of the total carcinogenic potency of PAHs showed that there are seasonal variations in TCP that follow the variations observed for PAH concentrations. TCPs that were estimated in this study are similar or slightly higher than those estimated at some other urban locations. The higher TCP values in this study were probably the result of slightly higher ambient levels of some carcinogenic PAHs, which is the consequence of a higher contribution of sources, such as wood and biomass burning. The high percentage contribution to the TCP shown by BaP regardless of the TEF scheme, season and fraction confirms that it is a good indicator for the carcinogenicity of the PAH mixture.

The relationship between total carcinogenic potencies determined for different particle fractions was analysed in this study as well as the relationship between TCPs and sums of PAH mass concentrations in different fractions. Stronger correlation of TCPs was found between PM_2.5_ and PM_1_ fractions than between PM_10_ and PM_2.5_ fractions. Linear correlations between TCPs in PM_1_ and PM_2.5–1_, as well as between PM_2.5_ and PM_10–2.5_ were weaker than those that were obtained with non-subtracted values, although statistically significant. Better correlation was found between smaller fractions—PM_1_ and PM_2.5–1_ and similar regression lines were obtained, regardless of the TEF scheme used. Regression analysis between TCP in PM_2.5_ and PM_10–2.5_ showed lower correlation coefficients. The highest correlation coefficient was obtained when TEFs by Nisbet and LaGoy were used. It was found that about 21–26% of carcinogenic potency of the PAH mixture in PM_10_ is present in the PM_2.5_ fraction (Figure 4a), whilst the ratio of total PAH mass concentration in PM_10_ and PM_2.5_ fraction was 29% (Figure S2a). The highest values were obtained when TEFs by Nisbet an LaGoy [27] were used, while TEFs by Muller [28] and Larsen and Larsen [29] gave almost identical results. Comparison of TCP in PM_2.5_ and PM_1_ (Figure 4b) showed that about 86% of carcinogenic potency of PM_2.5_ is present in the PM_1_ fraction, and the same result was obtained, regardless of the TEF scheme used. Comparison of ΣPAH in PM_2.5_ and PM_1_ showed that about 79% of ΣPAH in PM_2.5_ were present in the PM_1_ fraction (Figure S2b), which is lower than the value obtained for the corresponding comparison of TCPs between the same fractions. Overall, it seems that the increase in total PAH concentrations caused a higher increase of TCP in smaller particle fractions (PM_2.5_ and PM_1_) as compared to PM_10_.

Although the aforementioned linear regression methodology is often used for the comparison of mass concentrations of compounds in different fractions [35,46,55,56], it represents a new scientific approach for the comparison of carcinogenic activities of different particle fractions. We did not find a similar investigation carried out for carcinogenic potencies, especially with the PM_1_ fraction included. However, the parameters of regression lines indicated that the method is reliable for comparison of TCP in different fraction and it may be used in future studies. Analysis of the correlations between TCP and ΣPAH showed that the increase of ΣPAH of 10 ng m^−3^ in PM_10_ led to an increase in TCP between 2.3 and 3 ng m^−3^, while the same increase of ΣPAH in PM_2.5_ fraction led to an increase between 1.6 and 2.2 ng m^−3^. This all shows that the size of the particle has to be taken into consideration when the carcinogenic activity of PAHs is estimated.

Although estimations of PAH mixture carcinogenic potency using TEF are simple, they also have several disadvantages, so all the results obtained in this and similar studies have to be taken with caution. TEF values are based on the best available toxicological data from animal models. The calculated TEF values can vary within the dose range, which may be a problem because animal studies are performed with high doses and humans are exposed to lower concentrations [23]. For example, the TEF of DahA is around 5 at low dose and close to 1 at a higher dose based on local tumours induced by subcutaneous injection into mice [49]. Although there is an estimated proportion between human organism and mice, that as well contribute to the uncertainty of the results. The potential risk of PAH exposure may also be underestimated if the interactions of some PAHs are synergistic rather than additive. Larsen and Larsen [29], Bostrom et al. [23], and others pointed that studies on PAH mixtures have shown that they may interact metabolically in a number of different ways, resulting in synergistic, additive, or antagonistic effects, so nothing definitive can be concluded on the resulting tumorigenic actions of individual PAHs in complex mixtures. Due to the aforementioned reasons some authors used different approaches for risk estimation [57,58] Furthermore, only ten PAHs were measured in this study (due to the limitation of the analytical method), and only based on these ten PAHs we estimated the TCPs. Nitrated and oxygenated PAH compounds were not measured, which may have led to the underestimation of the full carcinogenic potential of PAH exposure. Samburova et al. [50] analysed the results of 13 projects in which 88 PAHs were measured in both the gas and particle phase. They concluded that the gas phase might contribute up to 30% to the sum of 88 PAHs and 16 EPA particle bound PAHs represent only 14.4% of the total gas and particle carcinogenic potency.

Regardless of the mentioned limitations, the PAH levels and estimated TCPs that were obtained in this study are found to be comparable with a lot of similar studies. Due to the fact that parallel measurements of PAHs in PM_10_, PM_2.5_, and PM_1_ fraction are relatively rare, the study will provide better insight to the carcinogenic potential of PAHs in different particle fractions. Detailed analysis of the contributions of individual PAHs to the sum of PAHs as well as the calculated ratios of PAHs between fractions have enabled identification of potential PAH sources. The methodology used for the comparison of carcinogenic activities of PAHs in different particle fractions may be used in future similar studies.

## 5. Conclusions

Measurements of ten PAHs in PM_10_, PM_2.5_, and PM_1_ particle fraction during four seasons of 2014 at an urban location in Zagreb, Croatia, were carried out in order to compare the PAH levels and their carcinogenic potency in different particle fractions and estimate the contribution of individual PAHs to the total carcinogenic activity of the PAH mixture. The estimations of total carcinogenic potency (expressed as a sum of BaP equivalent concentrations), and percentage contributions of individual PAHs to the total carcinogenic potency were calculated using three different toxic equivalency factor schemes. 

All of the measured PAHs as well as total PAH mass concentration and calculated TCPs showed pronounced seasonal variations with the lowest values during summer and the highest values during winter (heating season). Relative potency factors did not show significant seasonal variations, although in the PM_10_ fraction average RPF was highest in autumn, while in PM_2.5_ and PM_1_, it was highest in spring. 

The contributions of individual PAHs to the sum of PAH mass concentrations remained similar in all fractions and seasons. In the PM_10_ particle fraction, BghiP showed the highest percentage contributions, followed by BaP and BbF. In both PM_2.5_ and PM_1_ fractions, the highest contribution was shown by BghiP, followed by BbF and IP. DahA showed the lowest percentage contributions.

Contrary to what has been found for PM_10_, PM_2.5_, and PM_1_ fractions, the contributions of PAHs in subtracted fractions PM_10–2.5_ and PM_2.5–1_ varied significantly between seasons and fractions. For the overall measuring period in the PM_10–2.5_ fraction, the highest average percentage contributions showed BghiP and BaP. The high BbF contribution in summer together with relatively low contribution of BaP indicates traffic as a possible source of PAHs in coarse fraction. Regarding the fraction PM_2.5–1_, the differences between seasons were even more pronounced for BghiP. Very high contribution of BghiP in winter followed by low contribution of IP and BaP indicate petroleum combustion and traffic as a PAH source in the PM_2.5–1_ fraction. 

Comparison of ΣPAH in PM_2.5_ and PM_1_ fractions showed that about 79% of total PAHs in PM_2.5_ were present in the PM_1_ fraction, while the comparison of PAH sums in PM_10_ and PM_2.5_ showed that about 29% of total PAHs in PM_2.5_ were present in the PM_10_ fraction. 

Linear regression analysis and percentage contribution analysis of PAH mass concentrations showed that road traffic represented the most important source of PAHs in all fractions and throughout all seasons. Other sources (wood and biomass burning, petroleum combustion) were also present, especially during winter as a consequence of household heating. 

It was found that total carcinogenic potencies as well as the percentage contributions of PAHs to the TCP differed significantly for different TEF schemes applied (although a strong linear correlation was found between results). These results show that, in future research, the TEF scheme should be selected carefully taking into account the type of location, potential pollution sources, and atmospheric conditions. At locations with high levels of DahA (e.g., urban locations exposed to traffic or gasoline burning), the carcinogenic potency of PAHs could be overestimated if the TEFs of Nisbet and LaGoy [27] are applied. At locations with high levels of Flu (exposed to coal and biomass burning), the Larsen and Larsen [29] TEF scheme could cause overestimations of the TCP. However, some similarities were found regardless of the TEFs used.

The largest individual contributors to the TCP remained similar, regardless of the TEF scheme used for all particle fractions and seasons. In all cases, the highest contributions were shown by BaP, DahA, IP, and BbF, contributing together more than 90% of the carcinogenic potency. BaP showed the highest contribution to the total carcinogenic potency in all seasons and for all fractions. Due to the fact that BaP showed the highest contribution to the total carcinogenic potency in all particle fractions even when its concentrations in the air were low (it contributed to the sum of PAH mass concentrations only between 5% and 18%) it can be considered to be a good representative for assessing the carcinogenicity of the PAH mixture. 

Correlations between TCP and the sum of PAH mass concentrations showed that the increase of ΣPAH of 10 ng m^−3^ in PM_10_ led to an increase in TCP between 2.3 and 3 ng m^−3^, while the same increase of ΣPAH in PM_2.5_ fraction led to an increase of TCP between 1.6 and 2.2 ng m^−3^. This shows that the size of the particle has to be taken into consideration when the carcinogenic activity of PAHs is estimated. When comparing the carcinogenic potencies of PAHs in different fractions, a stronger correlation was found between PM_2.5_ and PM_1_ particle fractions than between fractions of PM_10_ and PM_2.5_. Between 21 and 26% of carcinogenic potency of PAHs present in PM_10_ belonged to the PM_2.5_ fraction. Comparison of TCP in PM_2.5_ and PM_1_ showed that about 86% of carcinogenic potency belonged to the PM_1_ fraction, regardless of the TEF scheme used. The aforementioned linear regression methodology applied in this study was found to be reliable for the comparison of carcinogenic activities of different particle fractions and it may be used in future studies. Analysis of correlations between TCP and ΣPAH showed that an increase of ΣPAH of 10 ng m^−3^ in PM_10_ led to an increase in TCP between 2.3 and 3 ng m^−3^, while the same increase of ΣPAH in the PM_2.5_ fraction led to an increase of TCP between 1.6 and 2.2 ng m^−3^.

## Figures and Tables

**Table 1:** Mass concentrations of PAHs (ng m^−3^) in PM_10_ particle fraction during different seasons of the year 2014 at an urban location in Zagreb.

PAH	Winter					Spring					Summer					Autumn				
	Mean	SD	γ_50_	Min	Max	Mean	SD	γ_50_	Min	Max	Mean	SD	γ_50_	Min	Max	Mean	SD	γ_50_	Min	Max
Flu	2.196	1.664	1.626	0.583	7.298	0.643	0.971	0.297	0.045	5.824	0.039	0.050	0.022	ND	0.219	0.323	0.382	0.212	ND	1.868
Pyr	1.944	1.395	1.576	0.623	5.982	0.570	0.804	0.271	0.033	4.634	0.029	0.032	0.017	ND	0.131	0.787	0.631	0.729	0.060	2.891
BaA	2.540	1.975	1.956	0.574	8.453	0.245	0.235	0.106	ND	0.803	0.035	0.034	0.027	ND	0.213	0.915	1.409	0.414	0.007	6.308
Chry	4.658	3.163	3.782	1.262	13.779	0.565	0.530	0.306	ND	1.953	0.042	0.044	0.029	ND	0.241	1.127	1.451	0.735	0.071	8.244
BbF	3.600	2.190	3.112	1.119	9.528	0.673	0.716	0.431	ND	2.673	0.089	0.086	0.075	ND	0.340	2.032	2.228	1.238	0.201	12.868
BkF	1.434	0.828	1.132	0.461	4.031	0.253	0.228	0.160	ND	0.718	0.035	0.035	0.030	ND	0.177	0.834	1.066	0.607	0.067	6.342
BaP	7.662	6.063	4.778	1.711	29.267	0.749	0.603	0.508	0.060	2.066	0.065	0.057	0.048	0.017	0.253	1.353	1.358	0.924	0.076	6.907
DahA	0.603	0.397	0.494	0.126	1.900	0.132	0.138	0.079	ND	0.443	0.007	0.008	0.008	ND	0.034	0.237	0.437	0.102	ND	2.733
BghiP	12.299	8.563	9.086	3.511	38.783	1.517	1.221	1.149	0.150	4.065	0.137	0.155	0.098	ND	0.772	2.656	2.284	2.024	0.151	10.105
IP	4.227	3.265	2.984	0.720	12.975	0.513	0.374	0.379	0.076	1.394	0.062	0.061	0.048	0.009	0.345	1.098	0.839	0.877	0.076	4.024
ΣPAH	41.163	27.958	33.439	11.819	124.386	5.859	4.708	4.030	0.630	15.700	0.539	0.481	0.435	0.055	2.259	11.363	10.944	8.057	8.057	0.788

ND—below detection limit; SD—standard deviation; γ_50_—median.

**Table 2:** Mass concentrations of PAHs (ng m^−3^) in PM_2.5_ particle fraction during different seasons of the year 2014 at an urban location in Zagreb.

PAH	Winter					Spring					Summer					Autumn				
	Mean	SD	γ_50_	Min	Max	Mean	SD	γ_50_	Min	Max	Mean	SD	γ_50_	Min	Max	Mean	SD	γ_50_	Min	Max
Flu	1.188	0.731	1.110	0.164	3.809	0.240	0.160	0.236	0.043	0.870	0.033	0.021	0.025	0.009	0.104	0.366	0.298	0.284	0.058	1.315
Pyr	1.135	0.803	0.994	0.211	5.024	0.210	0.139	0.203	0.022	0.540	0.023	0.020	0.018	0.001	0.107	0.356	0.291	0.269	0.066	1.516
BaA	1.039	0.738	0.828	0.076	3.461	0.112	0.100	0.079	ND	0.458	0.021	0.014	0.022	ND	0.063	0.270	0.280	0.152	0.037	1.061
Chry	2.212	1.478	1.674	0.202	6.717	0.265	0.193	0.230	ND	0.894	0.030	0.017	0.030	ND	0.078	0.433	0.439	0.285	0.070	2.067
BbF	2.332	1.225	1.837	0.340	5.780	0.463	0.343	0.371	ND	1.525	0.055	0.037	0.056	ND	0.224	0.940	0.834	0.593	0.130	3.759
BkF	1.328	0.667	1.323	0.303	2.721	0.206	0.154	0.152	ND	0.668	0.023	0.019	0.022	ND	0.125	0.354	0.299	0.216	0.038	1.243
BaP	2.393	1.395	2.115	0.365	7.549	0.270	0.202	0.237	0.013	0.919	0.027	0.023	0.026	ND	0.148	0.730	0.682	0.513	0.089	3.195
DahA	0.397	0.160	0.335	0.234	0.875	0.059	0.049	0.052	ND	0.241	0.002	0.008	ND	ND	0.036	0.122	0.145	0.072	ND	0.697
BghiP	6.920	3.372	6.709	1.273	19.615	0.795	0.422	0.724	0.196	1.838	0.165	0.068	0.153	0.062	0.345	1.903	1.540	1.153	0.254	6.203
IP	2.203	1.512	1.855	0.371	8.767	0.361	0.216	0.323	0.088	1.088	0.077	0.058	0.064	ND	0.395	0.715	0.536	0.470	0.096	2.468
ΣPAH	21.104	9.595	19.598	7.587	44.342	2.980	1.670	2.571	0.643	7.254	0.450	0.193	0.434	0.165	1.281	6.188	5.003	3.976	0.926	21.009

ND—below detection limit; SD—standard deviation; γ_50_—median.

**Table 3:** Mass concentrations of PAHs (ng m^−3^) in PM_1_ particle fraction during different seasons of the year 2014 at an urban location in Zagreb.

PAH	Winter					Spring					Summer					Autumn				
	Mean	SD	γ_50_	c	Max	Mean	SD	γ_50_	Min	Max	Mean	SD	γ_50_	Min	Max	Mean	SD	γ_50_	Min	Max
Flu	1.214	0.738	1.040	0.076	3.016	0.219	0.223	0.132	0.025	0.814	0.023	0.020	0.020	ND	0.070	0.204	0.196	0.144	ND	0.653
Pyr	1.210	1.024	0.822	0.132	4.313	0.151	0.111	0.112	0.031	0.534	0.024	0.019	0.020	ND	0.095	0.218	0.165	0.195	ND	0.677
BaA	0.873	0.505	0.751	0.107	2.175	0.089	0.042	0.082	0.034	0.199	0.025	0.011	0.027	ND	0.053	0.197	0.187	0.137	ND	0.793
Chry	1.825	1.181	1.499	0.227	5.816	0.176	0.087	0.166	0.049	0.401	0.032	0.017	0.033	ND	0.081	0.295	0.190	0.242	ND	0.907
BbF	2.293	0.947	2.346	0.419	4.134	0.332	0.166	0.313	0.069	0.707	0.059	0.030	0.057	ND	0.158	0.815	0.554	0.776	ND	2.277
BkF	1.113	0.512	1.058	0.194	2.319	0.127	0.062	0.122	0.032	0.285	0.027	0.013	0.027	ND	0.052	0.358	0.215	0.293	ND	0.775
BaP	2.228	1.298	1.986	0.452	6.413	0.205	0.128	0.186	0.035	0.517	0.030	0.015	0.028	ND	0.072	0.593	0.405	0.526	ND	1.773
DahA	0.317	0.165	0.302	ND	0.783	0.046	0.029	0.044	ND	0.143	0.003	0.009	ND	ND	0.033	0.105	0.168	0.051	ND	1.002
BghiP	4.199	2.346	3.527	0.250	9.951	0.650	0.322	0.610	0.168	1.381	0.116	0.071	0.105	ND	0.313	1.667	0.940	1.750	ND	3.498
IP	2.030	1.091	1.969	0.288	5.253	0.268	0.134	0.236	0.059	0.638	0.031	0.023	0.029	ND	0.090	0.616	0.401	0.570	ND	1.569
ΣPAH	17.274	8.443	16.632	4.203	34.709	2.262	1.091	2.216	0.655	4.750	0.372	0.186	0.351	0.030	0.848	5.069	2.918	4.829	0.000	12.198

ND—below detection limit; SD—standard deviation; γ_50_—median.

**Table 4:** The correlation between the mass concentrations of individual PAHs in particle fractions PM_1_, PM_2.5_, PM_2.5–1_, PM_10_, and PM_10–2.5_ for the whole sampling period (all correlation coefficients were significant at *p* < 0.05).

PAH	PM_2.5_ vs. PM_10_^1^			PM_1_ vs. PM_2.5_^2^			PM_1_ vs. PM_2.5–1_^3^			PM_2.5_ vs. PM_10–2.5_^4^		
	*a*	*b*	*r*	*a*	*b*	*r*	*a*	*b*	*r*	*a*	*b*	*r*
Flu	0.276	0.243	0.594	0.854	0.058	0.834	0.794	0.169	0.480	0.254	0.222	0.507
Pyr	0.346	0.153	0.630	0.843	0.049	0.728	0.695	0.157	0.640	0.253	0.181	0.512
BaA	0.178	0.222	0.492	0.670	0.067	0.880	0.916	0.146	0.608	0.161	0.256	0.406
Chry	0.267	0.339	0.580	0.743	0.040	0.917	0.889	0.259	0.443	0.244	0.401	0.513
BbF	0.280	0.551	0.512	0.732	0.205	0.821	0.698	0.467	0.460	0.174	0.583	0.340
BkF	0.398	0.246	0.547	0.703	0.078	0.864	0.692	0.260	0.430	0.088	0.319	0.133 *
BaP	0.182	0.432	0.659	0.833	0.060	0.912	0.879	0.487	0.424	0.180	0.485	0.583
DahA	0.254	0.089	0.512	0.594	0.036	0.672	0.381	0.068	0.366	0.238	0.097	0.411
BghiP	0.353	1.043	0.714	0.553	0.326	0.894	0.826	0.780	0.680	0.360	1.160	0.653
IP	0.336	0.356	0.687	0.767	0.109	0.915	0.989	0.404	0.521	0.293	0.474	0.563
ΣPAH	0.292	3.570	0.660	0.794	0.176	0.968	0.969	3.360	0.497	0.293	4.274	0.553

^1^ linear regression line (PAH)_PM2.5_ = *a* × (PAH)_PM10_ + *b*; ^2^ linear regression line (PAH)_PM1_ = *a* × (PAH)_PM2.5_ + *b*; ^3^ linear regression line (PAH)_PM1_ = *a* × (PAH)_PM2.5–1_ + *b*; ^4^ linear regression line (PAH)_PM2.5_ = *a* × (PAH)_PM10–2.5_ + *b*; * the correlation was not significant.

**Table 5:** Total carcinogenic potency (TCP) and relative factor potency (RPF) of PAHs bounded to particle fractions PM_10_, PM_2.5_, and PM_1_ during 2014 at a Zagreb urban site using toxic equivalency factors (TEFs) of Nisbet and LaGoy [27], Muller [28], and Larsen and Larsen [29].

Particle Fraction	Season	Nisbet and LaGoy (1992)				Muller (1997)				Larsen and Larsen (1998)			
		TCP (ng m^−3^)		RPF		TCP (ng m^−3^)		RPF		TCP (ng m^−3^)		RPF	
		Mean	SD	Mean	SD	Mean	SD	Mean	SD	Mean	SD	Mean	SD
PM_10_	Winter	12.031	8.729	1.635	0.162	9.235	7.014	1.226	0.046	9.690	7.289	1.292	0.060
	Spring	1.598	1.376	2.109	0.463	1.020	0.826	1.362	0.105	1.107	0.886	1.486	0.133
	Summer	0.125	0.100	1.986	0.857	0.090	0.076	1.396	0.195	0.096	0.080	1.496	0.255
	Autumn	3.067	3.902	2.223	0.608	1.966	2.093	1.473	0.132	2.077	2.212	1.564	0.163
	Overall	4.287	6.750	1.985	0.616	3.142	5.208	1.363	0.158	3.310	5.438	1.458	0.196
PM_2.5_	Winter	5.111	1.913	2.479	1.019	3.345	1.612	1.494	0.277	3.608	1.713	1.618	0.316
	Spring	0.689	0.448	2.908	1.606	0.423	0.274	1.744	0.683	0.464	0.293	1.945	0.834
	Summer	0.058	0.069	2.078	1.364	0.044	0.036	1.628	0.287	0.050	0.039	1.827	0.364
	Autumn	1.592	1.355	2.300	0.686	1.041	0.905	1.456	0.143	1.118	0.955	1.574	0.184
	Overall	1.898	2.310	2.447	1.233	1.237	1.600	1.576	0.412	1.335	1.715	1.735	0.506
PM_1_	Winter	4.503	2.174	2.205	0.567	3.049	1.599	1.421	0.146	3.268	1.691	1.533	0.182
	Spring	0.525	0.280	2.750	0.804	0.319	0.178	1.621	0.186	0.352	0.193	1.801	0.236
	Summer	0.063	0.059	1.852	0.951	0.045	0.025	1.462	0.195	0.049	0.028	1.565	0.249
	Autumn	1.337	1.176	2.179	0.964	0.861	0.590	1.460	0.170	0.923	0.632	1.569	0.208
	Overall	1.639	2.153	2.246	0.884	1.090	1.473	1.490	0.189	1.171	1.571	1.615	0.242

SD—standard deviation.

**Table 6:** Total carcinogenic potencies in different particle fractions determined by other authors.

Study	Type of Location	TEF	Particle Fraction	TCP (ng m^−3^)
Bari et al. [30]	residential site	* Nisbet and LaGoy 1992	PM_10_	2.7
Pooltawee et al. [9]	Phayo Province, Northern Thailand	Larsen and Larsen 1998 * Nisbet and LaGoy 1992	coarse, fine, ultrafine	June: 0.183 March: 29.04
Masiol et al. [4]	industrial	* Nisbet and LaGoy 1992	PM_2.5_	1.9
Hanedar et al. [21]	residential area	* Nisbet and LaGoy 1992	TSP	2.164
Petry et al. [51]	urban	* Nisbet and LaGoy 1992		0.906
Chang et al. [52]	traffic	* Nisbet and LaGoy 1992	PM_10_	0.89
Akyüz et al. [36]	industrial	* Nisbet and LaGoy 1992	PM_2.5_ PM_2.5-10_	winter: 22.5050 summer: 0.7223 winter: 1.2410 summer: 0.2684
Kozielska et al. [10]	regional background urban background traffic point	Nisbet and LaGoy 1992 Durant et al. 1996 Willett et al. 1997	PM_1_	Heating non-heating 5.85 4.50 18.46 5.29 106.0 15.46
Agudelo-Castañeda et al. [2]	urban area	* Nisbet and LaGoy 1992	PM_1_	winter: 0.662 summer: 0.272
Delgado-Saborit et al. [47]	traffic roadside	* Nisbet and LaGoy 1992 WHO 1999 EPAQS 1999		5.8–7.8
Callen et al. [53]	urban	Larsen and Larsen 1998	PM_10_	1.82
Manoli et al. [39]	urban	* Nisbet and LaGoy 1992 Malcolm and Dobson 1994 Durant et al. 1996	PM_10_ PM_2.5_	winter: 1.4 summer: 0.25 winter: 1.4 summer: 0.2
Khan et al. [31]	semi-urban area	* Nisbet and LaGoy 1992	PM_2.5_	0.572
Masiol et al. [32]	rural background	* Nisbet and LaGoy 1992	PM_10_	1.7
Jung et al. [49]	outdoor	* Nisbet and LaGoy 1992	gas phase, PM_2.5_	0.450
Majewski et al. [54]	urban	* Nisbet and LaGoy 1992	PM_1_	3.38
Jakovljević et al. [41]	rural urban residential urban traffic urban industrial	* Nisbet and LaGoy 1992	PM_10_	winter summer 3.765 0.079 3.663 0.079 5.039 0.080 5.173 0.182
This study	urban residential	Nisbet and LaGoy 1992	PM_10_ PM_2.5_ PM_1_	4.287 1.898 1.639
This study	urban residential	Muller 1997	PM_10_ PM_2.5_ PM_1_	3.142 1.237 1.090
This study	urban residential	Larsen and Larsen 1998	PM_10_ PM_2.5_ PM_1_	1.458 1.735 1.615

* Nisbet and LaGoy modified method (1992).

## Supplementary Materials

The following are available online at http://www.mdpi.com/1660-4601/15/11/2485/s1, Table S1: The toxic equivalence factors (TEFs) used in the study, Table S2: Correlation between total carcinogenic potencies (TCPs) and relative factor potencies (RPFs) estimated using different TEF schemes. The calculation is made for the whole sampling period. (TCP_N_—Nisbet and LaGoy [27], TCP_M_—Muller [28], TCP_L_—Larsen and Larsen [29]), Table S3: Average percentage contribution (%) of PAHs to the total carcinogenic potency—PM_10_ particle fraction, Table S4: Average percentage contribution (%) of PAHs to the total carcinogenic potency—PM_2.5_ particle fraction, Table S5: Average percentage contribution (%) of PAHs to the total carcinogenic potency—PM_1_ particle fraction, Table S6: Average values of temperature (TEMP), relative humidity (RH), pressure (PRESS) and the total amount of precipitation for all measuring periods of 2014 in the study area, Figure S1 The percentage contributions of individual PAHs to the sum of the measured PAHs in (a) winter; (b) spring; (c) summer; (d) autumn, in PM_2.5–1_, PM_10–2.5_, PM_10_, PM_2.5_ andPM_1_ particle fraction, Figure S2: Correlation between the sum of PAH mass concentrations (ΣPAH) in particle fractions (a) PM_2.5_ and PM_10_; (b) PM_1_ and PM_2.5_ for the whole measuring period, Figure S3: Total carcinogenic potency (TCP) of PAHs bounded to particle fraction (a) PM_10_, (b) PM_2.5_ and (c) PM_1_ during 2014 at a Zagreb urban site using toxic equivalency factors (TEFs) of Nisbet and LaGoy (TCP_N_) [27], Muller (TCP_M_) [28] and Larsen and Larsen (TCP_L_) [29], Figure S4: Relative potency factor (RPF) of PAHs bounded to particle fraction (a) PM_10_, (b) PM_2.5_ and (c) PM_1_ during 2014 at a Zagreb urban site using toxic equivalency factors (TEFs) of Nisbet and LaGoy (RPF_N_) [27], Muller (RPF_M_) [28] and Larsen and Larsen (RPF_L_) [29].
