# Supplementary material for: Carcinogenic Potency of Airborne Polycyclic Aromatic Hydrocarbons in Relation to the Particle Fraction Size
Source: Int J Environ Res Public Health. 2018 Nov 7;15(11):2485. doi: 10.3390/ijerph15112485 (PMC6266409; doi:10.3390/ijerph15112485)
Supplement: Supplementary file 1 [file ijerph-15-02485-s001.pdf]

International Journal of
Environmental Research and Public Health

MDPI

Table S1. The toxic equivalence factors (TEFs) used in the study [27-29].

PAH	Toxic equivalence factors (TEFs)		
	Nisbet and LaGoy (1992)	Muller (1997)	Larsen and Larsen (1998)
Flu	0.001		0.050
Pyr	0.001	0.000	0.001
BaA	0.100	0.014	0.005
Chry	0.010	0.026	0.030
BbF	0.100	0.110	0.100
BkF	0.100	0.037	0.050
BaP	1.000	1.000	1.000
DahA	5.000	0.890	1.100
BghiP	0.010	0.012	0.020
IP	0.100	0.067	0.100

Table S2. Correlation between total carcinogenic potencies (TCPs) and relative potencies factor (RPFs) estimated using different TEF schemes. The calculation is made for the whole sampling period. (TCP_N- Nisbet and LaGoy [27]) TCP_M –Muller [28], TCP_L – Larsen and Larsen [29]).

Particle fraction	Relationship	Linear correlation coefficient	Probability
PM_{10}	TCP_N vs TCP_M	0.9826	<0.00001
	RPF_N vs RPF_M	0.8937	<0.00001
	TCP_N vs. TCP_L	0.9847	<0.00001
	RPF_N vs. RPF_L	0.8929	<0.00001
	TCP_M vs. TCP_L	0.9998	<0.00001
	RPF_M vs. RPF_L	0.9870	<0.00001
$PM_{2.5}$	TCP_N vs TCP_M	0.9732	<0.00001
	RPF_N vs RPF_M	0.8727	<0.00001
	TCP_N vs. TCP_L	0.9763	<0.00001
	RPF_N vs. RPF_L	0.8658	<0.00001
	TCP_M vs. TCP_L	0.9998	<0.00001
	RPF_M vs. RPF_L	0.9965	<0.00001
PM_1	TCP_N vs TCP_M	0.9921	<0.00001
	RPF_N vs RPF_M	0.8728	<0.00001
	TCP_N vs. TCP_L	0.9929	<0.00001
	RPF_N vs. RPF_L	0.8542	<0.00001
	TCP_M vs. TCP_L	0.9999	<0.00001
	RPF_M vs. RPF_L	0.9490	<0.00001

Table S3. Average percentage contribution (%) of PAHs to the total carcinogenic potency - PM_{10} particle fraction.

TEF	PAH	Overall	Winter	Spring	Summer	Autumn
Nisbet and LaGoy (1992)	Flu	0.03	0.02	0.06	0.03	0.02
	Pyr	0.03	0.02	0.05	0.03	0.04
	BaA	2.48	2.06	1.43	3.48	2.92
	Chry	0.37	0.40	0.34	0.33	0.41
	BbF	5.75	3.23	4.48	7.40	7.89
	BkF	2.23	1.35	1.50	2.93	3.13
	BaP	54.12	61.72	49.51	57.08	47.73
	DahA	29.43	26.66	37.67	21.79	32.06
	BghiP	1.06	1.04	1.02	1.10	1.08
	IP	4.50	3.51	3.94	5.81	4.72
Muller (1997)	Flu	0.00	0.00	0.00	0.00	0.00
	Pyr	0.00	0.00	0.00	0.00	0.00
	BaA	0.47	0.38	0.30	0.62	0.57
	Chry	1.32	1.38	1.31	1.10	1.50
	BbF	8.62	4.73	7.26	10.21	12.29
	BkF	1.13	0.67	0.83	1.38	1.65
	BaP	74.23	81.67	73.79	72.89	68.36
	DahA	8.29	6.41	10.86	6.78	9.28
	BghiP	1.74	1.66	1.81	1.66	1.83
	IP	4.21	3.12	3.85	5.37	4.52
Larsen and Larsen (1998)	Flu	1.82	1.28	3.11	1.97	0.98
	Pyr	0.04	0.02	0.05	0.03	0.05
	BaA	0.16	0.13	0.10	0.21	0.19
	Chry	1.43	1.51	1.40	1.19	1.64
	BbF	7.36	4.08	6.14	8.70	10.55
	BkF	1.44	0.86	1.04	1.74	2.10
	BaP	69.66	77.59	67.77	68.47	64.53
	DahA	9.54	7.51	12.41	7.67	10.75
	BghiP	2.71	2.62	2.75	2.60	2.86
	IP	5.85	4.41	5.22	7.42	6.33

Table S4. Average percentage contribution (%) of PAHs to the total carcinogenic potency - $PM_{2.5}$ particle fraction.

TEF	PAH	Overall	Winter	Spring	Summer	Autumn
Nisbet and LaGoy (1992)	Flu	0.05	0.02	0.04	0.10	0.03
	Pyr	0.04	0.02	0.04	0.07	0.03
	BaA	2.33	2.05	1.74	3.83	1.67
	Chry	0.44	0.44	0.45	0.57	0.28
	BbF	7.24	4.97	7.60	10.29	6.16
	BkF	3.06	2.51	3.41	4.08	2.29
	BaP	45.59	44.37	41.89	48.44	47.51
	DahA	29.41	40.25	36.70	4.82	35.94
	BghiP	2.23	1.34	1.52	4.80	1.25
	IP	9.63	4.02	6.62	22.99	4.86
Muller (1997)	Flu	0.00	0.00	0.00	0.00	0.00
	Pyr	0.00	0.00	0.00	0.00	0.00
	BaA	0.45	0.45	0.38	0.62	0.34
	Chry	1.59	1.80	1.80	1.68	1.07
	BbF	11.11	8.72	13.05	12.85	9.99
	BkF	1.60	1.50	1.93	1.72	1.25
	BaP	63.70	68.55	61.79	54.91	69.34
	DahA	9.29	12.24	11.87	2.18	10.92
	BghiP	3.59	2.51	2.64	6.98	2.22
	IP	8.67	4.23	6.54	19.06	4.87
Larsen and Larsen (1998)	Flu	2.71	1.64	2.90	4.54	1.82
	Pyr	0.05	0.03	0.05	0.07	0.04
	BaA	0.15	0.15	0.12	0.20	0.11
	Chry	1.67	1.92	1.86	1.74	1.15
	BbF	9.19	7.34	10.65	10.47	8.42
	BkF	1.96	1.87	2.35	2.08	1.57
	BaP	58.36	63.45	56.21	49.16	64.38
	DahA	10.43	13.90	13.17	2.31	12.37
	BghiP	5.00	3.87	3.94	8.75	3.43
	IP	10.49	5.82	8.75	20.68	6.72

Table S5. Average percentage contribution (%) of PAHs to the total carcinogenic potency - PM_1 particle fraction.

TEF	PAH	Overall	Winter	Spring	Summer	Autumn
Nisbet and LaGoy (1992)	Flu	0.03	0.03	0.05	0.04	0.02
	Pyr	0.03	0.03	0.03	0.04	0.03
	BaA	2.68	1.94	1.97	5.04	1.70
	Chry	0.43	0.41	0.38	0.60	0.32
	BbF	7.84	5.39	6.91	11.29	7.80
	BkF	3.54	2.59	2.66	5.06	3.85
	BaP	49.26	48.00	39.52	57.80	51.36
	DahA	28.58	36.19	41.40	9.58	27.53
	BghiP	1.52	0.92	1.49	2.06	1.64
	IP	6.08	4.51	5.58	8.48	5.74
Muller (1997)	Flu	0.00	0.00	0.00	0.00	0.00
	Pyr	0.00	0.00	0.00	0.00	0.00
	BaA	0.49	0.41	0.43	0.80	0.33
	Chry	1.51	1.59	1.55	1.79	1.07
	BbF	11.56	8.96	12.09	14.12	11.15
	BkF	1.73	1.44	1.56	2.14	1.79
	BaP	67.23	71.05	62.43	65.97	69.24
	DahA	9.19	10.38	13.21	4.62	8.69
	BghiP	2.47	1.65	2.79	2.88	2.62
	IP	5.82	4.53	5.95	7.69	5.12
Larsen and Larsen (1998)	Flu	2.17	2.02	3.14	2.21	1.32
	Pyr	0.04	0.04	0.05	0.05	0.03
	BaA	0.16	0.13	0.14	0.27	0.11
	Chry	1.61	1.69	1.62	1.94	1.15
	BbF	9.74	7.55	9.91	12.06	9.48
	BkF	2.17	1.81	1.90	2.71	2.26
	BaP	62.34	66.09	56.40	61.98	64.60
	DahA	10.26	11.84	14.67	4.81	9.87
	BghiP	3.80	2.55	4.17	4.48	4.05
	IP	7.72	6.27	8.00	9.50	7.12

Table S6. Average values of temperature (TEMP), relative humidity (RH), pressure (PRESS) and the total amount of precipitation for all measuring periods of 2014 in the study area.

	TEMP (°C)	RH (%)	PRESS (hPa)	Precipitation (L m^{-2})
Winter	5.0	84.2	998.7	6.8
Spring	13.1	70.2	997.8	5.7
Summer	21.5	72.5	998.0	9.9
Autumn	13.3	82.1	1003.9	13.1

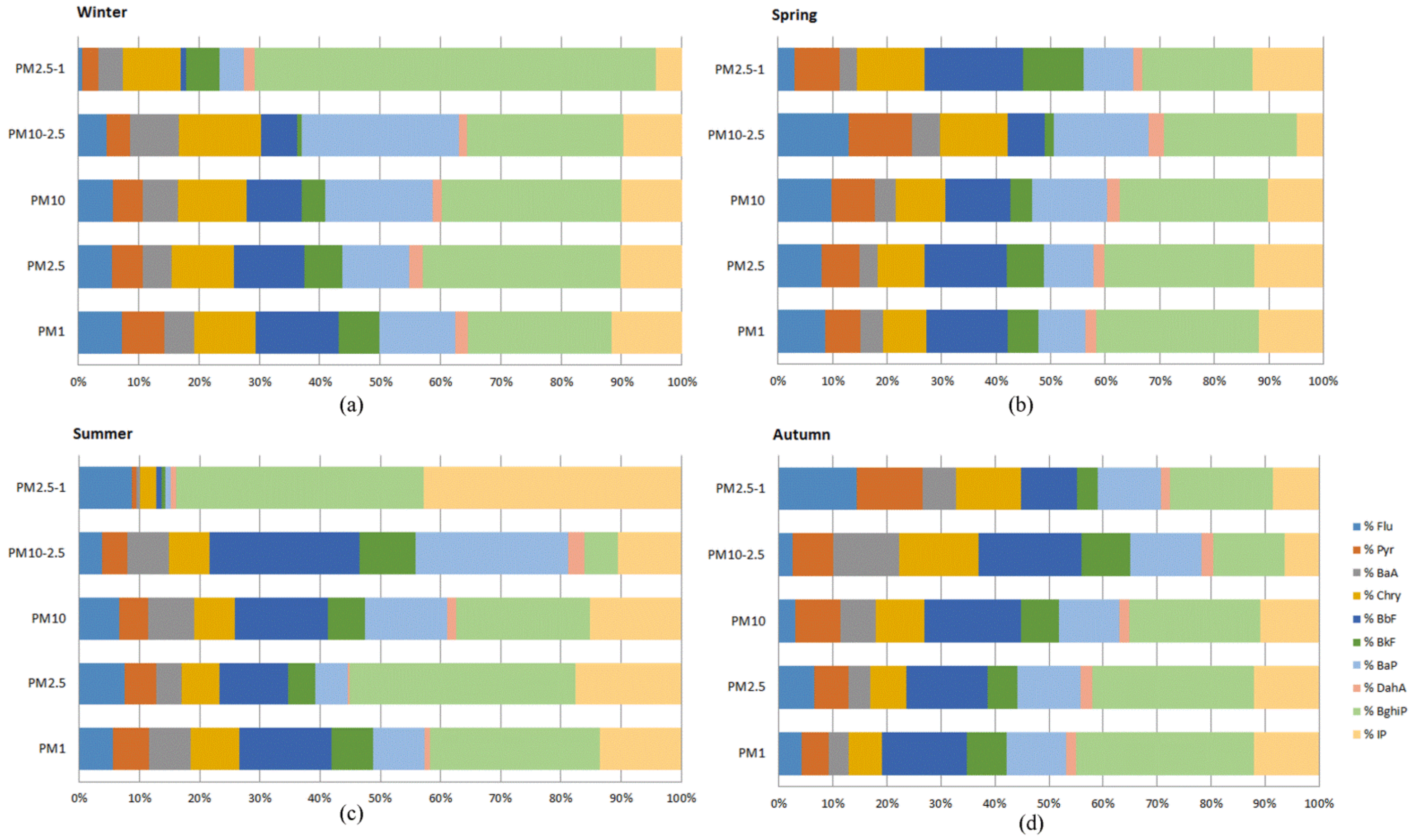

Figure S1. The percentage contributions of individual PAHs to the sum of the measured PAHs in (a) winter; (b) spring; (c) summer; (d) autumn, in $PM_{2.5-1}$, $PM_{10-2.5}$, PM_{10}, $PM_{2.5}$ andPM_1 particle fraction.

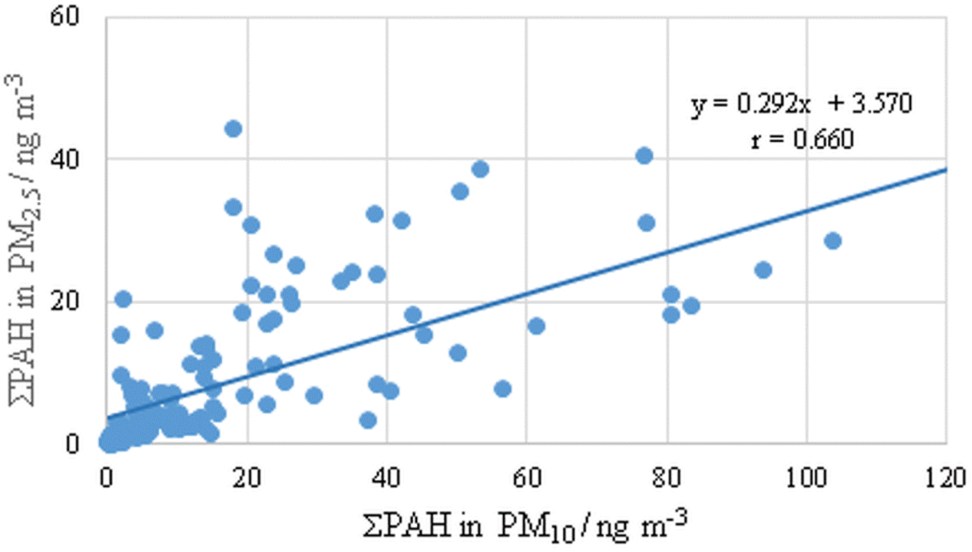

(a) $PM_{2.5}$ vs. PM_{10}

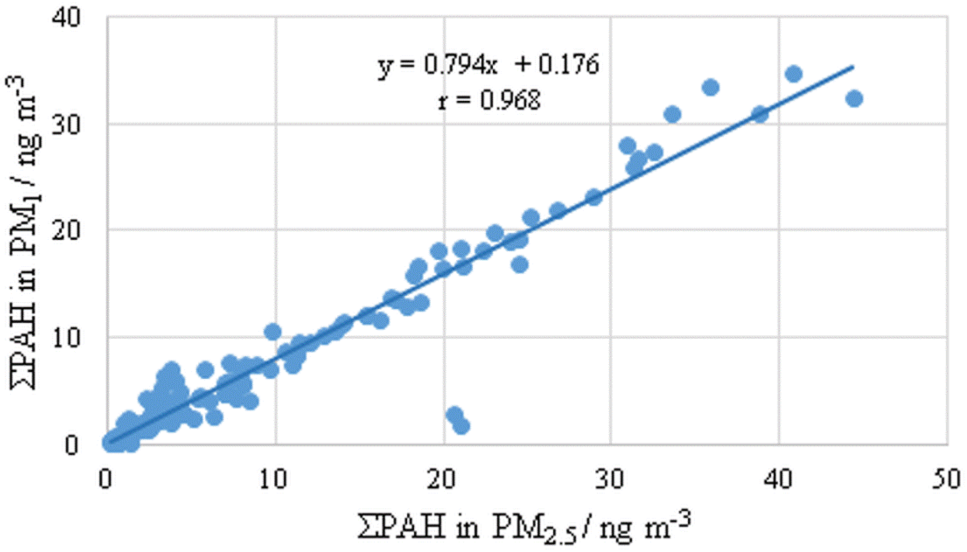

(b) PM_1 vs. $PM_{2.5}$

Figure S2. Correlation between the sum of PAH mass concentrations (ΣPAH) in particle fractions (a) $PM_{2.5}$ and PM_{10}; (b) PM_1 and $PM_{2.5}$ for the whole measuring period.

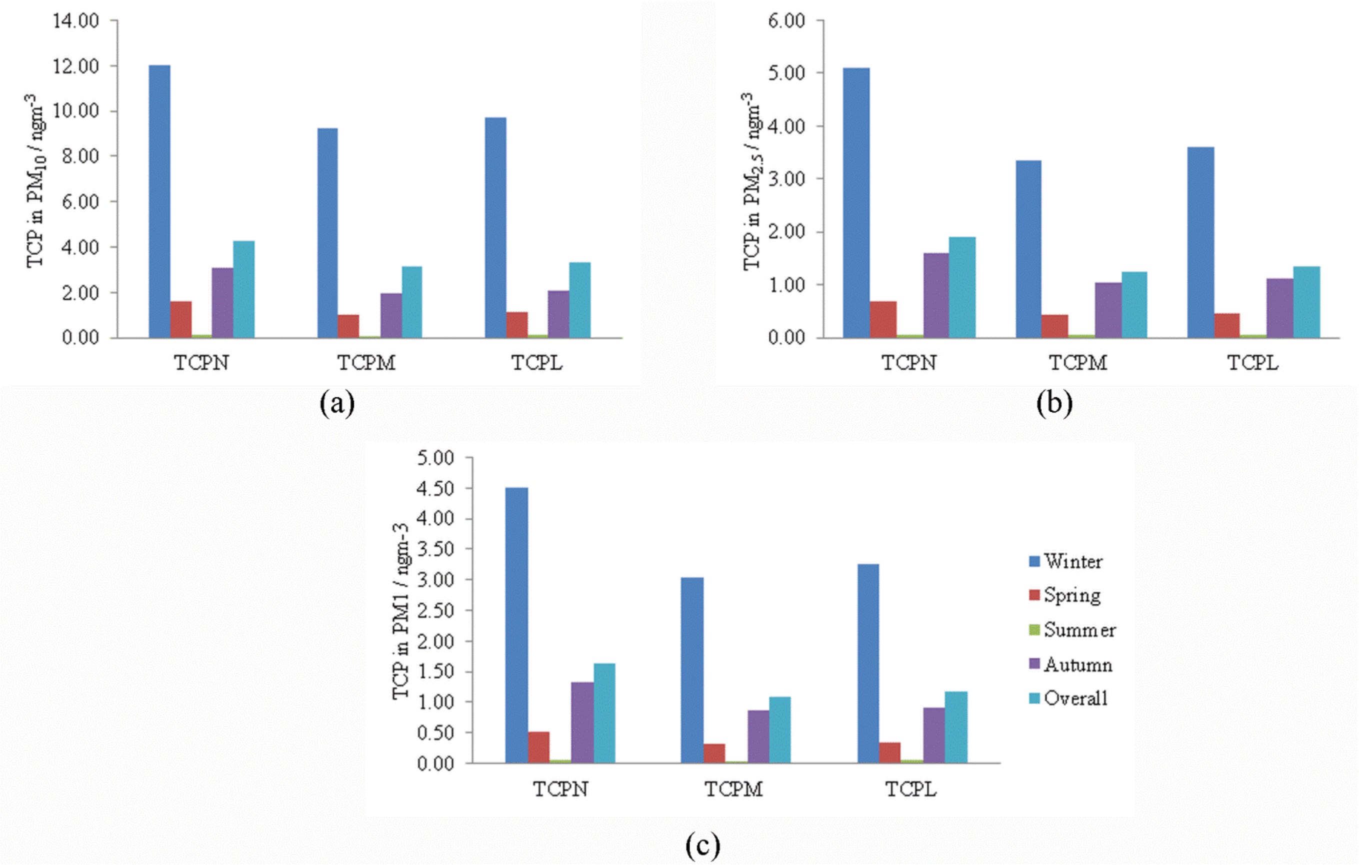

Figure S3. Total carcinogenic potency (TCP) of PAHs bounded to particle fraction (a) PM_{10}, (b) $PM_{2.5}$ and (c) PM_1 during 2014 at a Zagreb urban site using toxic equivalency factors of Nisbet and LaGoy (TCP_N) [27], Muller (TCP_M) [28] and Larsen and Larsen (TCP_L) [29].

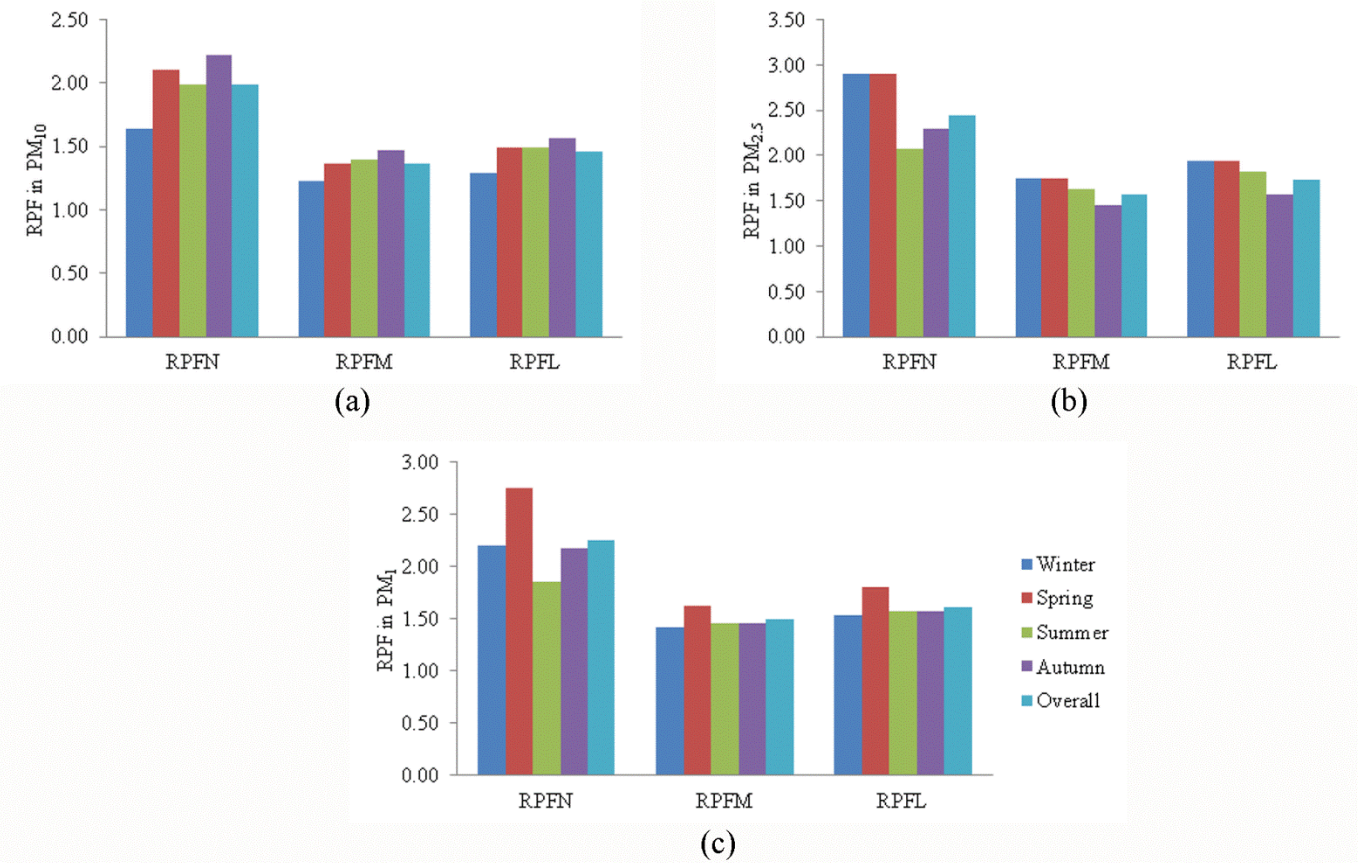

Figure S4. Relative potency factor (RPF) of PAHs bounded to particle fraction (a) PM_{10}, (b) $PM_{2.5}$ and (c) PM_1 during 2014 at a Zagreb urban site using toxic equivalency factors of Nisbet and LaGoy (RPF_N) [27], Muller (RPF_M) [28] and Larsen and Larsen (RPF_L) [29].
